# DSM Nosology Changes in Neuropsychological Diagnoses through the Years: A Look at ADHD and Mild Neurocognitive Disorder

**DOI:** 10.3390/bs7010001

**Published:** 2016-12-29

**Authors:** Anne R. Carlew, Andrea L. Zartman

**Affiliations:** 1 Department of Psychology, University of North Texas, 1155 Union Cir, Denton, TX 76203, USA; anne.carlew@unt.edu; 2 Veterans Affairs North Texas Health Care System, Mental Health, 4500 S. Lancaster Road, Dallas, TX 75216, USA

**Keywords:** neuropsychology, nosology, mild cognitive impairment, attention-deficit hyperactivity disorder

## Abstract

This article discusses the evolution of modern neuropsychology as a field and the concomitant changes in the Diagnostic and Statistical Manual of Mental Disorders (DSM). Themes in neuropsychology through the years will be highlighted alongside discussion of how neuropsychologists and neuropsychological research have influenced and have been influenced by the DSM. The DSM 5 attention-deficit/hyperactivity disorder and mild neurocognitive disorder will be used as examples to reflect the evolution of the disorders in relation to neuropsychology and the DSM. In particular, recent criticism and research regarding the nosology of both disorders and future directions will be presented in the context of neuropsychology and DSM. Finally, influence regarding changes to the DSM 5 on neuropsychology in clinical decision making, test selection, and diagnosis will be discussed.

## 1. Introduction

The Diagnostic and Statistical Manual for Mental Disorders (DSM) has been a guiding force for psychologists and psychiatrists in the conceptualization of psychological disorders. Changes in social and scientific thinking over the years have not only shaped the manual but also scientific/clinical investigation and conceptualization into what it is today. Specifically, for neuropsychologists, advancements in genetic testing and neuroimaging have added to our knowledge of neurodevelopmental and neurocognitive disorders. However, we have been slow to embrace this knowledge and incorporate it into everyday practice. The present article will briefly review the history of the key neuropsychological disorders in the DSM and outline the most recent changes salient for neuropsychologists. Special focus on the diagnostic evolution of attention-deficit/hyperactivity disorder (ADHD) and mild neurocognitive disorder (mild NCD) in context of DSM will be highlighted. Finally, current limitations and future directions for both aforementioned diagnoses and the field will be discussed.

## 2. Evolution of Neuropsychology and Its Diagnoses in the DSM

Neuropsychology as a field was first born out of the quest to associate behaviors with specific brain regions. The early works of Broca [1] and Wernicke [2] are classic examples of this approach. While cognitive testing was developed by educational psychologists in the early 1900s, the first generation of neuropsychologists developed psychometric techniques to quantify behavior and localize brain lesions based on test performance in the mid-1900s [3].

Neuropsychology has historically been thought of as most relevant to the diagnoses in the DSM 5’s now “Neurodevelopmental Disorders” and “Neurocognitive Disorders” chapters. This encompasses intellectual disabilities, communication disorders, autism spectrum disorders, attention-deficit hyperactivity disorder, specific learning disorders, motor disorders, delirium, mild neurocognitive disorder, and major neurocognitive disorder [4]. However, significant bodies of research have also highlighted the neuropsychological components of psychiatric disorders such as schizophrenia [5] and depression [6]. For the purposes of this review, we have chosen to focus on one diagnosis from the “Neurodevelopmental Disorders” chapter, ADHD, and one diagnosis from the “Neurocognitive Disorders” chapter, mild NCD, to illustrate changes applicable to neuropsychological diagnoses in the DSM as a whole.

The original DSM came on the heels of World War II in 1952 and was heavily influenced by scientific investigation and discovery of the time. The manual was mainly divided into two sections capturing diagnoses with some known biological etiology and disorders thought to be psychogenic in nature. The neuropsychological diagnoses reflected in the DSM 5 today were captured in a section titled “Disorders Caused by or Associated with Impairment of Brain Tissue or Function”. These disorders were meant to represent conditions in which changes to brain tissue were apparent and were further delineated into acute and chronic. Although the concept of ADHD has been discussed and researched since its first mention in 1798 by Sir Alexander Crichton, there was no mention of the disorder in this volume. In relation to neurocognitive disorders, there was no differentiation of symptom severity. As such, the concept of mild NCD was non-existent. Instead, it was subsumed under the precursor of today’s major neurocognitive disorder (major NCD) captured in the volume’s “Chronic Brain Syndromes” with specifiers of varying etiologies such as infection and trauma. In general, these diagnostic categories were descriptive in nature and did not offer criteria for diagnosis [7].

The large number of head injuries from the war in addition to the growth of psychology itself through the publication of the DSM provided the platform for rapid growth and development of neuropsychology as a field [3]. The principle of double dissociation (i.e., two related mental processes are shown to function independently of each other) gave rise to the profile analysis of test scores that point to distinct brain regions [8]. Neurologists and neurosurgeons depended on these early neuropsychologists for neuropsychological test results in addition to EEGs and X-rays for localization [3].

In 1968, the original DSM was revised to align more closely with international diagnostic thinking, the International Classification of Diseases (ICD). In the DSM II, the nosology for cognitive disorders changed to “Organic Brain Syndromes” (OBS’s) subdivided into psychotic and non-psychotic. In a similar fashion to the original DSM, the diagnostic categories offered little in the way of diagnostic criteria outside of the names of the disorders themselves. Today’s major NCD was captured in the diagnosis “Psychoses Associated with Organic Brain Syndromes”. In this conceptualization, patients were diagnosed as psychotic if functioning was limited enough to interfere with daily functioning. Patients were diagnosed with non-psychotic OBS if their cognitive functioning was not deteriorated enough to interfere with daily functioning [9].

Today’s ADHD was first included in this edition labeled “hyperkinetic reaction of childhood” in the Behavior Disorders of Childhood and Adolescence Chapter. Prior to the publication of the DSM II, the Oxford International Study Group of Child Neurology recommended the label “minimal brain dysfunction” [10]. Similarly, the National Institute of Neurological Diseases and Blindness assigned a task force to delineate the symptoms of minimal brain dysfunction which culminated in the following:

…refers to children of near average, average or above average general intelligence with certain learning or behavioural disabilities ranging from mild to severe, which are associated with deviations of function of the central nervous system. These deviations may manifest themselves by various combinations of impairment in perception, conceptualisation, language, memory and control of attention, impulse or motor function.

[11] (p. 9)

The DSM II task force used the nomenclature hyperkinetic reaction of childhood in part due to the growing criticism that the concept of minimal brain dysfunction was too broad, and hyperactivity was one of the most salient aspects of the disorder [12]. Consequently, this label described hyperactive and inattentive behaviors, but did not offer criteria, cutoffs, or age of onset. While there was some mention of cognitive symptoms at this time, the focus was still on the behavioral aspects of the disorder [10].

This period was somewhat of a shift for neuropsychology. Neuropsychologists such as Jackson and Luria strayed from the localization perspective of their predecessors and proposed a functional model in which behavior is a product of several different brain areas working together whereby a disruption to any one area may disrupt an entire system [3]. The advent of neuroimaging and the CT scanner in the 1970s perpetuated this shift from localization to function. Neuropsychologists began to focus more on descriptions of cognitive functioning and behavioral outcomes, despite using the same tests developed for localization. Cognitive psychologists also began investigating cognitive constructs such as sustained attention and visual memory at this time. Thus, the second wave of neuropsychological tests were developed to measure these constructs [3].

With the DSM III in 1980 came the advent of the multi-axial system and greatly expanded diagnostic criteria, a change that was unique to the field of psychology and less in line with the ICD [13]. Its publication ushered out the psychoanalyst, syndrome-based approach to the diagnosis and treatment of mental disorders and ushered in the scientific era of German psychiatrist Emil Kraepelin, again more closely aligning the field as a whole with physical medicine. This change came about via dissatisfaction with the unclear diagnoses, low agreement between professionals in the field, and disagreement regarding what constitutes normal and abnormal mental health. Thus, the neo-Kraepelinians advocated epidemiological research and descriptive work [14]. The Research Diagnostic Criteria (RDC), a collection of diagnostic criteria developed to allow for consistent diagnosis in psychiatric research across countries, was published in the late 1970s and influenced many of the diagnostic criteria published in the DSM III. This edition of the DSM dropped the descriptive paragraphs associated with disorders and added empirically-based diagnostic criteria [14].

The chapter entitled “Organic Brain Disorders” was divided into organic brain disorder (if of known etiology) and organic brain syndrome (if of unknown etiology). This is the first we see of symptom severity being considered in diagnostic differentials. While the terms mild cognitive impairment (MCI) or mild NCD are still not explicitly stated, the diagnostic label of dementia was reserved for patients who had lost functional independence. Atypical or mixed organic brain syndrome was used for patients with a milder presentation who were still functionally independent. Mild, moderate, and severe specifiers were given based on the patient’s level of functional impairment. However, these specifiers tended to be arbitrary rather than from a psychometric standpoint [13].

In relation to ADHD, Paul Wender’s work on minimal brain dysfunction syndrome shifted the conversation toward an attention-based, brain arousal hypothesis [15] in the interim between DSM II and DSM III. He was largely influenced by the works of Sykes and Douglas who investigated the nuanced attentional differences in hyperactive children compared to controls. For instance, they pioneered the concept of sustained attention deficits despite intact attentional mechanisms over more brief periods of time [16]. Thus, the term attention deficit disorder (ADD) with or without hyperactivity was introduced in the third version of the DSM in the chapter “Disorders Usually First Evident in Infancy, Childhood, or Adolescence” with specification of age-of-onset criteria before age 7. In this edition, the diagnostic criteria for ADD with hyperactivity or without hyperactivity was based solely on the symptom of hyperactivity [13]. Thus, the symptom of impulsivity was mistakenly placed in the diagnostic criteria for ADD without hyperactivity [17]. The revision of the DSM III, the DSM III-R, dropped the subtypes of ADD due to lack of empirical evidence for the subtypes and added specifiers for severity (i.e., mild, moderate, or severe) [18].

During this time, neuropsychology continued to evolve along with the field of psychology as neuroimaging methods with more spatial and temporal fidelity were produced [3]. Subsequent versions of the DSM built on the DSM III’s trend toward descriptive empiricism and greater specificity [14]. A surge of epidemiological studies allowed the addition of more descriptive information to subsequent DSMs. These DSM editions also incorporated information from studies of genetics, pathophysiology, and neuroimaging. In the DSM IV, the chapter for organic brain disorders was renamed to “Delirium, Dementia and Amnestic or Other Cognitive Disorders” to correct the implication that non-organic mental disorders did not have some biological basis [19,20]. DSM III’s ADD was renamed attention-deficit/hyperactivity disorder (ADHD) and was divided into inattentive, hyperactive, and combined subtypes. Despite these changes, many clinicians continued to follow Barkley’s suggested criteria rather than the DSM IV as once again the research did not align with the DSM IV criteria [21]. Per Barkley’s criteria, ADHD was primarily a disorder of executive dysregulation in the domains of behavioral inhibition, working memory, regulation of motivation, and motor control rather than simply of attention and hyperactivity [22].

## 3. Changes to Neuropsychological Diagnoses from DSM IV-TR to DSM 5

The change from the DSM IV-TR to the DSM 5 continued APA’s quest to align itself more closely with the current research in molecular biology, psychometrics, and the cognitive and affective neurosciences. Its structure was also proposed to match the upcoming ICD-11 [14]. Overall, the DSM 5 reflects the continuation of a significant shift in conceptualization of psychological disorders to more closely understand and identify the contributing factors and underlying etiology. Perhaps no other field could benefit more from this than neuropsychology. The changes from DSM IV-TR to DSM 5 were significant in that they reflect the field’s current understanding of the dimensionality of various developmental, psychological, and neurological disorders. It also more closely reflects the developmental nature of some disorders such as ADHD. Furthermore, the DSM 5 incorporated diagnostic criteria based on the previous decade’s research, notably in the DSM 5’s ADHD and mild neurocognitive disorder [4]. It is to these classifications we turn to examine the evolving nosology of neuropsychological mental disorders related to both development and acquired etiologies and to understand where we still fall short.

## 4. Changes to Attention-Deficit/Hyperactivity Disorder

Some of the more noticeable changes to the DSM 5 were to the diagnostic criteria for ADHD. Barkley reviewed the state of diagnostic criteria for ADHD and made recommendations for adjustments from the current research [21]. Several these suggestions were incorporated into the DSM 5 in keeping with the committee’s aforementioned vision. ADHD was moved from the “Disorders Usually First Diagnosed in Infancy, Childhood, or Adolescence” chapter to one entitled “Neurodevelopmental Disorders” to capture its developmental nature and cognitive symptoms [4]. Barkley reported the expression of ADHD symptoms changed over the course of the lifespan (e.g., fewer hyperactive symptoms expressed with age/hyperactive symptoms expressed differently with age). He also highlighted the arbitrary nature of the cut-off age of onset as 7 [21]. Thus, the onset age was raised to 12. Furthermore, diagnostic descriptions and criteria were altered to apply more appropriately to adults in response to criticism that the DSM IV-TR criteria lacked sensitivity to adults with ADHD and growing recognition that the syndrome persists into adulthood [23], another reflection of the developmental nature of the disease [22]. For example, criteria for diagnosis are met with five symptoms of the disease instead of six to reflect the persistent but changing symptom clusters typically seen as one becomes older. Additionally, instead of the requirement from the DSM IV-TR that symptoms be present in multiple environments and impair functioning, the DSM 5 requires symptoms to be present in two or more environments but impaired functioning need not be present, only “…clear evidence that the symptoms interfere with, or reduce the quality of, social, academic, or occupational functioning,” a change that has been met with criticism. The DSM 5 also now requires clinicians to specify severity [4]. Overall, the changes to ADHD in the DSM 5 have been met with uncertainty [24,25,26].

## 5. Addition of Mild Neurocognitive Disorder

A significant change for neuropsychology to the DSM came with the final formal recognition of mild neurocognitive disorder (mild NCD) as a separate diagnostic category. The disorder, which most closely reflects what researchers and clinicians have long called MCI, was added in response to the growing acknowledgement that early identification and treatment of neurodegenerative disorders results in better outcomes (i.e., slower disease progression) [27]. Petersen and colleagues first introduced the concept of MCI in 1997 in an attempt to capture a group of patients who had memory impairment despite other preserved cognitive skills, without resulting functional impairment [27]. This group of patients, by definition, does not meet criteria for dementia and are thus at risk for being overlooked for treatment. In previous editions of the DSM, this group had only been captured in unspecified, “catch-all” diagnostic categories such as “Cognitive Disorder Not Otherwise Specified”. In Petersen’s original paper, he noted approximately 10%–15% of patients went on to develop dementia, particularly those with specific biomarkers (i.e., one or more ε4 alleles at the polymorphic APOE locus) [27]. Since that time, the MCI construct has undergone considerable changes from different groups to become broader and more differentiated. For example, an International Consensus Working Group proposed a diagnostic structure which included type (memory vs. non-memory) and number of domains affected (single vs. multiple) [28]. However, the document does not discuss the value or significance of biomarkers in making the diagnosis. Similarly, the APA’s proposed mild NCD, which was fashioned most closely after the 2003 Key Symposium criteria [28], focuses on syndromal aspects of the entity and requires significant (typically 1 to 1.5 standard deviations) cognitive decline in at least one of six stated domains including: complex attention, executive functioning, language, perceptual-motor, learning and memory, or social cognition. In keeping with the traditional definition of dementia, the diagnosis requires preserved functional abilities, but does specify “…a level of cognitive decline that requires compensatory strategies and accommodations to help maintain independence and perform activities of daily living” [4].

While the DSM 5 does not include genetic or imaging information in the actual diagnostic criteria for mild NCD and its etiologies, it does require the clinician to specify probable vs. possible, which incorporates this information. For example, for a diagnosis of probable mild NCD due to Alzheimer’s disease to be made, family history and/or evidence of the presence of the Alzheimer’s gene must be present. In the absence of this evidence, the disorder is specified as “possible”. The manual also includes information on diagnostic markers that may or may not be readily available to the diagnosing clinician. For example, the DSM 5 includes information on genetic mutations that may be present in frontotemporal dementia (FTD) and specifies characteristic patterns of atrophy frequently observed in FTDs [4].

## 6. Current Limitations

The DSM has evolved over the years from an ambiguous document of poorly delineated classifications to a flexible, descriptive manual based in empiricism. While it has certainly come a long way, the document is not without its limitations. Some of these limitations are a product of the document itself, while others reflect limitations in current scientific knowledge [29]. Analogously, the field of neuropsychology suffers from several limitations which in turn hinder optimal diagnostic sensitivity and specificity [3].

First, while the DSM 5 is the first to structure itself to reflect the dimensional nature of cognitive and psychological disorders, it still falls short of capturing the true heterogeneity of disorders themselves [30]. Second, it fails to strongly incorporate interdisciplinary research, which may be important to supporting diagnosis and understanding the clinical presentation [31]. However, the diagnosis of possible versus probable major NCD and mild NCD does incorporate findings from genetics, neuroimaging, and biology, which is an improvement upon previous versions. Third, many clinicians and researchers argue that the language of the DSM 5 is vague [32]. The change from “clinically significant impairment” to “interfering with functioning” serves to make an already vague concept even vaguer. For example, clinicians often run into the question of whether impairment is due to true neurological disturbance or the result of contributing factors such as co-morbid psychological distress, poor sleep, or medication effects. Building on this train of thought, it is unclear from a neuropsychological perspective what quantitatively is considered a “low score”. While some studies may consider scores below the 16th percentile impaired (Heaton), others do not consider impairment until the 5th percentile (Wechsler). Clinicians and researchers would benefit from operationalization of quantitative and qualitative categories of functioning. Furthermore, it is unclear whether more than one score within the same domain should be impaired to represent true impairment. While the DSM 5 does offer some guidance on the level of impairment required (i.e., one standard deviation below the mean), it does not cite hard evidence that this is the best practice [4].

Neuropsychology continues to lag behind scientific discovery in several areas. First, neuropsychologists continue to rely upon measures developed for the localization of injury or assessment of a cognitive construct. Many neuropsychologists fail to incorporate more recently developed measures focused on functional status, which have a higher ecological and predictive validity [3]. As the DSM continues to place more emphasis on functional status in disorders such as mild NCD, it will be important for neuropsychology as a field to keep up with developments as the concept of “functional impairment” becomes more refined. Furthermore, the worth of neuropsychological testing is enhanced as our ability to accurately predict real-world functioning increases. This becomes even more important as the referral questions have historically changed from localization to questions of capacity, the return to school/work, and differential diagnosis for treatment [3]. Second, research should continue to focus on the development of age, ethnicity, and education stratified normative samples. Currently, widely published normative samples beyond Caucasian and African American ethnicities are rare. Several studies have highlighted the importance of appropriate normative data, particularly when diagnosis is made from scores on neuropsychological testing (e.g., mild and major NCD) [33].

## 7. Future Directions

Several authors have commented on the DSM 5 and made recommendations for future versions of the diagnostic criteria for ADHD and mild NCD. For example, Barkley has argued for the inclusion of concentration deficit disorder (CDD), a related but distinct concept to ADHD [17]. Previously named sluggish cognitive tempo, Barkley has recommended CDD for inclusion in the DSM to capture a group of individuals who experiences cognitive symptoms of sluggishness, fogginess, and confusion along with motor symptoms of hypoactivity and lethargy, with onset in childhood. Research has been conducted on this construct since the 1980s which has separated it from the ADHD diagnosis, but it has yet to be included in the DSM and currently only exists as a research entity [17]. In addition, further research is needed to investigate the effects of diagnostic changes and additions to current clinical practice. There is some research to suggest the new criteria for diagnosing adult ADHD does not significantly improve over the DSM IV-TR criteria enough to outweigh the potential negative effects [25]. Regarding etiology, more recent work has postulated fluctuations in performance in individuals with ADHD could be due to ineffective suppression of the default mode network which interferes with current tasks [34]. Further research may inform etiological questions as well as intervention efforts.

The addition of mild NCD has received extensive commentary. First, researchers have pointed out the DSM 5 has more strictly delineated the construct of MCI [35]. However, some have expressed concern the current mild NCD will result in a higher number of false-negatives, especially when issues such as non-credible performance or other factors of reduced engagement are not taken into account. Indeed, research has shown diagnostic criteria for mild NCD captures only half of the individuals who would have been diagnosed with MCI [36]. Second, mild NCD was modeled after previous MCI research, which was conducted in geriatric populations, while mild NCD can be diagnosed at any age. Thus, further research is needed to validate the construct in a more age-diverse sample [37]. However, the DSM 5 mild NCD improves over previous versions of the conceptualization in several ways. For example, it eliminated the criteria of memory impairment for any type of dementia. Instead, it specifies and includes all domains of cognition including language, memory, executive function, visuospatial function, complex attention, and social cognition [4]. Its structure also allows for progressively more specific classification of dysfunction based on available evidence [38]. Thus, research may benefit by grouping together mild NCD diagnoses by possible or probable etiology rather than grossly combining the entire diagnosis.

Neuropsychological research would benefit from efforts in two main areas. First, developing more functionally based neuropsychological tests would allow neuropsychologists to more precisely answer referral questions based on competency and prediction of independent functioning [39]. Furthermore, it may help future editions of the DSM 5 more specifically delineate functional impairment, similar to its current delineation of cognitive impairment more than 1–1.5 standard deviations below the mean. Second, the development of multivariate normative datasets including ethnicities beyond African American and Caucasian would also enhance sensitivity and specificity, and prevent misunderstanding of symptoms presentations [33].

## 8. Conclusions

In conclusion, the *DSM* as a document and neuropsychology as a field have evolved over time in response to scientific discovery, clinical need, and social and political pressures. Both fall short of taking full advantage of the more recent advances in research and technology, as evidence by the current diagnostic criteria of mild NCD and ADHD. These diagnoses continue to be contingent upon syndromal aspects and distinct categories rather than incorporating pathological markers of disease, when available, and promoting true dimensional approaches to diagnosis and classification. Neuropsychologists have been slow to adapt to changes in the field whereby referral questions are increasingly geared toward functional capacity and continue to rely upon measures originally designed to localize disease or measure cognitive constructs that may or may not translate to real-world deficits. Further research efforts are need to address these limitations.
